# Outbreak of Pulmonary Tuberculosis in a Chinese High School, 2009–2010

**DOI:** 10.2188/jea.JE20120216

**Published:** 2013-07-05

**Authors:** Yirong Fang, Lijie Zhang, Chunyu Tu, Dongqing Ye, Robert Fontaine, Huilai Ma, Jiahu Hao, Lijun Fu, Xijun Ying, Qifeng Chen, Yong Wang, Huihui Liu, Bao-Ping Zhu

**Affiliations:** 1 School of Pubulic Health, Anhui Medical University, Hefei, China; 2 Department of Infectious Disease, Shaoxing Center for Disease Control and Prevention, Shaoxing, China; 3 Chinese Field Epidemiology Training Program, Chinese Center for Disease Control and Prevention, Beijing, China; 4 Department of Global Health, United States Centers for Disease Control and Prevention, Atlanta, Georgia, United States; 5 Department of Infectious Disease, Shenzhou Center for Disease Control and Prevention, Shenzhou, China

**Keywords:** epidemiology, outbreak, students, tuberculosis

## Abstract

**Background:**

In February 2009, a high school student was diagnosed with sputum-smear positive pulmonary tuberculosis (TB). One year later, 2 other students in the same grade developed sputum-smear positive TB.

**Methods:**

We used tuberculin skin testing (TST), chest radiography, sputum smear, and symptomatology for case identification. We defined latent TB infection (LTBI) as a TST induration of 15 mm or larger, probable TB as a chest radiograph indicative of TB plus productive cough/hemoptysis for at least 2 weeks or TST induration of 15 mm or larger, and confirmed TB as 2 or more positive sputum smears or 1 positive sputum smear plus a chest radiograph indicative of TB.

**Results:**

Of students in the same grade as the primary case-student, 26% (122/476) had LTBI and 4.8% (23/476) had probable/confirmed TB. Of teachers, 43% (18/42) had LTBI and none had probable/confirmed TB. Sharing a classroom with the primary case-student increased risk for LTBI (rate ratio = 2.5; 95% CI: 1.9–3.4) and probable/confirmed TB (rate ratio = 17, 95% CI: 7.8–39). Of students with LTBI in February 2009 who refused prophylaxis, 50% (11/22) had probable/confirmed TB in April 2010.

**Conclusions:**

This TB outbreak was likely started by delayed diagnosis of TB in the case-student and was facilitated by lack of post-exposure chemoprophylaxis. Post-exposure prophylaxis is strongly recommended for all TST-positive students.

## INTRODUCTION

An estimated 1.3 million Chinese (1 in 1000) develop new tuberculosis (TB) annually, which accounts for 1 of 7 new TB cases globally.^1^ TB epidemics in China are characterized by large numbers of infected people, symptomatic patients, deaths, rural patients, and patients with drug-resistant TB.^2^ The crowded living conditions in dormitories and the close proximity of students in classrooms facilitate transmission of TB in schools^3^; consequently, TB outbreaks frequently occur in Chinese schools.^4^ However, little is understood about the transmission dynamics and risk factors of TB in Chinese schools.

In March 2010, 2 cases of sputum smear-positive (SS+) pulmonary TB were identified in a high school (31 classes, 1574 students) in the eastern Chinese province of Zhejiang (2010 population: 54 426 891). We investigated this outbreak to determine the proper treatment of case-students, identify gaps in TB prevention and control, and offer recommendations for TB control strategies in Chinese schools.

## METHODS

### Study design

We retrospectively reviewed medical records and interviewed doctors and nurses at the school’s health clinic. To collect information on disease onset and exposure history, we conducted in-person interviews of the case-students or telephoned their parents. We reviewed administrative records to evaluate measures taken by the county Center for Disease Control and Prevention (county CDC) to control this outbreak.

### Tuberculin skin testing

The county CDC staff performed tuberculin skin testing (TST), using standard procedures. Briefly, a trained nurse injected intradermally 0.1 ml (2 IU) of purified protein derivative (PPD) produced from bacille Calmette-Guérin (Chendu Institute of Biological Products, Chendu, China) into the inner surface of the left forearm. An experienced physician measured the transverse induration (in mm) at the TST site 72 hours after injection.^5^^,^^6^

### Case definition

Latent TB infection (LTBI) was defined as a TST induration with a diameter of 15 mm or larger.^2^ Probable TB was defined as a chest radiograph indicative of TB plus a productive cough/hemoptysis lasting for at least 2 weeks or a TST induration with a diameter of 15 mm or larger. Confirmed TB was defined as 2 or more positive sputum smears or 1 positive sputum smear plus a chest radiograph indicative of TB.

### Case finding

A self-administered questionnaire was used to screen for suspicious symptoms (ie, productive cough or night sweats lasting for ≥2 weeks) among students and the family members of SS+ case-students. Those with a TST induration of 15 mm or larger or suspicious TB symptoms were screened by chest radiography.^7^ If the chest radiograph was inconclusive, a computed tomography (CT) scan of the suspicious lesions was obtained by an experienced physician.^7^ For those with abnormal results on chest radiography or CT, or those with symptoms indicative of TB, up to 3 unconcentrated sputum specimens (night, morning, and spot) were examined by microscopy.^7^

### Statistical analysis

Statistical analysis was performed using the SPSS statistical package (version 11.0). Categorical variables were analyzed using the χ^2^ test. A *P* value of 0.05 or less was considered to be statistically significant.

### Ethical considerations

This investigation was a response to a public health emergency and was exempted by the Institutional Review Board of the China Centers for Disease Control. To protect the confidentiality of the participant, all data were deidentified and kept confidential.

## RESULTS

Our investigation identified 4 SS+ case-students. The primary case-student developed symptoms (including productive cough, sore throat, lack of appetite, and weight loss) in mid-November 2008. He sought medical care multiple times over 3 months at various hospitals and clinics but was misdiagnosed and received antibiotic treatment. His symptoms worsened, leading to difficulty sleeping. On February 10, 2009, he went to a hospital and was diagnosed as having SS+ TB with widespread foci of infection in the lung and a cavitary lesion in the lung field. During the 3 months from symptom onset to confirmation of SS+ TB diagnosis, the primary case-student had continuously attended school. After the TB diagnosis, he was excluded from school and hospitalized.

The county CDC subsequently launched TST screening of the students (*n* = 46) and teachers (*n* = 17) who shared the classroom with the primary case-student and found that 61% (28/46) of students and 53% (9/17) of teachers had LTBI (TST induration ≥15 mm). Free prophylaxis with isoniazid was arranged for those students and teachers. One student with LTBI took 1 dose of isoniazid and then declined further treatment; the other 27 students and 9 teachers declined prophylactic treatment, fearing hepatotoxicity (Table 1).

**Table 1.** Post-exposure chemoprophylaxis of students and teachers with a TST induration ≥15 mm during a tuberculosis outbreak in a high school in Zhejiang Province, China (2009–2010)

Prophylaxis	2009 (*n* = 37^a^)		2010 (*n* = 126)	
	
	*n*	%	*n*	%
Completed recommended 6-month regimen	0	—	70	56
Quit	1^b^	2.7	6^c^	4.8
No prophylaxis, due to adverse effects	0	—	5^d^	4.0
Declined prophylaxis	36^e^	97.3	45^e^	36

Abbreviation: TST, tuberculin skin testing.

^a^When tested again in April 2010, 11 had developed probable/confirmed tuberculosis and 12 still had latent tuberculosis infection.

^b^1 student took 1 dose and then quit, fearing hepatotoxicity.

^c^2 students quit due to abnormalities in liver enzymes and 4 teachers quit due to concerns regarding hepatotoxicity.

^d^4 students had abnormal liver enzymes and 1 had fever.

^e^Declined drug due to concerns regarding hepatotoxicity.

On March 24 and 30, 2010, 2 additional students in the same grade as the primary case-student were diagnosed with SS+ TB after exhibiting TB symptoms and seeking medical care. Although they were in the same grade as the primary case-student, their classrooms differed from that of the primary case-student, and neither reported any direct contact with the primary case-student. Subsequently, the county CDC conducted another round of TST among 476 students in this grade and 42 teachers who taught in this grade. A fourth SS+ case-student was identified among students who shared the classroom of the primary case-student. She was asymptomatic but had a positive TST, an abnormal chest radiograph, and was SS+ on 3 consecutive sputum specimens (Figure).

**Figure.**
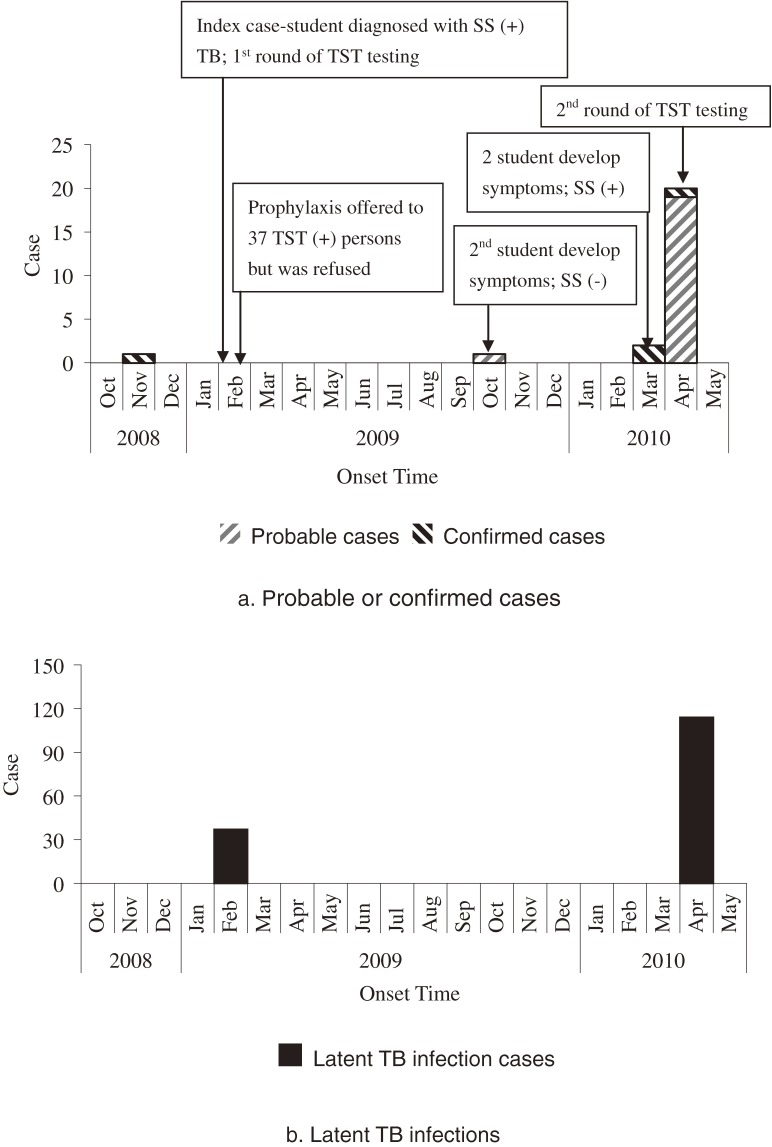
Timeline of probable/confirmed tuberculosis (TB) cases and latent TB infections in a high school in Zhejiang Province, China (2009–2010). SS, sputum smear; TST, tuberculin skin testing.

The county CDC ordered mandatory isolation and treatment for all probable and confirmed case-students identified during the second round of screening. Free prophylactic treatment was arranged for all students and teachers with LTBI. Still, only 56% (70/126) of those students and teachers completed the recommended 6-month regimen (Table 1).

In total, we identified 164 cases (including 20 probable and 4 confirmed TB cases and an additional 140 LTBI cases) between February 2009 and April 2010. Of 24 persons with probable/confirmed TB, 4 were symptomatic (including lethargy, lack of appetite, productive cough, and fever). The LTBI rate was lower among students (26%) than among teachers (43%) (rate ratio [RR] = 0.60, 95% CI: 0.41–0.88); 4.8% of the students and none of the teachers developed probable/confirmed TB (Table 2).

**Table 2.** Latent TB infection and probable/confirmed TB among students (and their teachers) in the same grade as the index case in a high school in Zhejiang Province, China, 2009–2010

Group	*N*	Latent TB Infection			Probable/Confirmed TB		
	
		*n*	Rate (%)	RR (95% CI)	*n*	Rate (%)	RR (95% CI)
Students	476	122	26	0.60 (0.41–0.88)	23^a^	4.8	∞ (0.57–∞)
Teachers	42	18	43		0	0	

Abbreviations: RR, rate ratio; TB, tuberculosis.

^a^Excluding index case.

Among 476 students in the same grade as the primary case-student, sharing a classroom was significantly associated with higher risk of LTBI (RR = 2.5, 95% CI: 1.9–3.4) and probable/confirmed TB (RR = 17, 95% CI: 7.8–39). The rate of latent TB and probable/confirmed TB did not differ significantly by sex in the primary case-student’s classroom, whereas in other classrooms, girls had a higher LTBI rate than boys (RR = 0.64, 95% CI: 0.45–0.92) (Table 3).

**Table 3.** Latent TB infection and probable/confirmed TB among students in the same grade as index case, by exposure (Zhejiang Province, China, 2009–2010)

Exposure	Total No.	No. tested	Latent TB Infection			Probable/Confirmed TB		
	
			*n*	Rate (%)	RR (95% CI)	*n*	Rate (%)	RR (95% CI)
Same classroom as index case	46	46	26	57	2.5 (1.9–3.4)^a^	15	33	17 (7.8–39)^a^
Boys	22	22	13	54	0.92 (0.55–1.5)	10	42	1.8 (0.74–4.5)
Girls	24	24	13	59	Ref	5	23	Ref
Different classroom from index case^b^	430	428	96	22	Ref	8	1.9	Ref
Boys	227	225	40	18	0.64 (0.45–0.92)	5	2.2	1.5 (0.36–6.2)
Girls	203	203	56	28	Ref	3	1.5	Ref
Residential status								
Boarding	235	233	63	32	1.1 (0.81–1.4)	11	4.7	0.95 (0.43–2.1)
Commuting	241	241	59	29	Ref	12	4.9	Ref

Abbreviations: RR, rate ratio; TB, tuberculosis.

^a^Compared with students in different classroom from index case.

^b^Index case received TB diagnosis in February 2009.

Thirty-nine (79%) of the 46 students and 9 (53%) of the 17 teachers who were tested in February 2009 were tested again in April 2010. Of the 22 students who had LTBI in February 2009, 50% (11/22) developed probable/confirmed TB and 41% (9/22) still had LTBI in April 2010. Of the 5 teachers who had LTBI in February 2009, none developed into probable or confirmed cases, whereas 3 (60%) still had LTBI. Of the 17 students who were TST-negative in February 2009, 24% (4/17) had developed into probable/confirmed cases and 53% (9/17) had developed LTBI. Of the 4 teachers who were TST-negative in February 2009, none developed probable/confirmed TB and 1 (25%) developed LTBI (Table 4).

**Table 4.** Results of screening in February 2009 and April 2010 of students in the same classroom as index case (Zhejiang Province, China, 2009–2010)

Screening result in February 2009		No. screened	Screening result in April 2010			

			No. of Cases		Infection Rate (%)	
	
			Probable/Confirmed TB	Latent TB Infection (TST ≥15 mm)	Probable/Confirmed TB	Latent TB Infection (TST ≥15 mm)
TST-positive (≥15 mm)	Student	22	11	9	50	41
	Teacher	5	0	3	0	60
						
TST-negative (<15 mm)	Student	17	4	9	24	53
	Teacher	4	0	1	0	25

Abbreviations: TST, tuberculin skin testing; TB, tuberculosis.

## DISCUSSION

During this outbreak, the primary case-student developed TB symptoms in November 2008. His SS+ pulmonary TB was not diagnosed for 3 months, during which he continued to attend school. Sharing a classroom with this case-student was associated with increased risk of LTBI and probable/confirmed TB. Subsequent SS+ case-students were identified quickly and were not associated with increased risk of infection. Hence, delayed diagnosis of TB in the primary case-student likely started this outbreak, and lack of post-exposure chemoprophylaxis likely contributed to TB spread.

TB transmission is airborne; outbreaks often occur among people living in crowded, closed spaces for extended periods of time (eg, school students). Risk factors for school TB outbreaks include constant contact with SS+ students, insufficient ventilation, and delayed diagnosis.^8^^,^^9^ Early detection, timely isolation and treatment of patients, and post-exposure prophylaxis are the only effective prevention measures. This outbreak exposed failures in all 3 aspects of TB control. First, despite repeatedly seeking medical care and presenting with typical TB symptoms, it took approximately 3 months to diagnose TB in the primary case-student.

Second, the primary case-student stayed in school for 3 months without treatment, causing widespread transmission. According to the Regulation on Tuberculosis Prevention and Control in Schools by the Ministry of Health of China,^10^ a student diagnosed with TB must be excluded from school for mandatory treatment for at least 2 months and cannot resume school activities until 3 consecutive negative sputum smears (separated by an interval of at least 1 month) have been collected. In addition, lesions in the lung field must have healed for at least 2 months, as shown by chest radiography, and the student must have normal findings on at least 3 physician-certified follow-ups. Additionally, all close contacts must be screened and treated as necessary. This strict regulation places a heavy burden on students and their families, especially when students in high school are preparing for the all-important national college entrance examination. Consequently, many high schools choose to ignore TB symptoms in students. This might partially explain why the primary case-student remained in school while being symptomatic for 3 months. Published evidence, however, suggests that patients with SS+ TB usually need only 2 to 3 weeks of isolation and that those with SS− TB require only 4 to 7 days if they receive the correct drugs.^11^ Therefore, the strict regulations in China are unwarranted at best and counterproductive at worst. Third, although taking isoniazid once daily for 6 months was associated with minimal hepatotoxicity,^12^ almost all the students and teachers who were TST-positive after the first round of screening, and nearly 30% of students and over 40% of teachers who were TST-positive after the second round of screening, refused prophylactic treatment, fearing hepatotoxicity. This false public perception might have been due to misleading reports in Chinese journals, which described post-prophylaxis hepatotoxicity without qualifying patient age or prior liver conditions.^13^

In a prospective study in Angola, 31.8% of LTBI cases progressed to active TB, and 9.4% of uninfected children had developed active TB at 6 months of follow-up.^14^ In our investigation, among students who shared a classroom with the primary case-student, were TST-positive in February 2009, and did not receive prophylactic treatment, half developed probable or confirmed TB by April 2010. Moreover, among TST-negative students in February 2009, nearly a quarter had developed probable or confirmed TB in April 2010. These results suggest that, after a long period of exposure (in this case >3 months) to SS+ patients in a crowded setting (in this case a classroom), the risk of developing TB (based on our definition) is high.

This investigation had several limitations. First, after TB was diagnosed in the primary case-student in February 2009, the county CDC only conducted TST among students and teachers sharing a classroom with the primary case-student; moreover, after 3 additional SS+ case-students were later identified, the county CDC still only conducted TST among students of the same grade as the primary case-students. Because TB is transmitted through the air, other, unidentified cases may have been present in this school, and those cases might have been undercounted. Second, sputum cultures were not collected; hence drug resistance analysis could not be done. Third, without isolates of *Mycobacterium tuberculosis* we were unable to confirm by genotyping that all TB patients in this outbreak were infected with the same strain.

To ensure early identification of TB, we recommend that healthcare providers in China routinely obtain a sputum smear and chest radiograph from all patients presenting with a cough of 2 or more weeks’ duration. Public health authorities in China should re-evaluate and revise, if necessary, the strict regulations on compulsory exclusion from school for 2 months or longer for students diagnosed with TB. In addition, public health and clinical professionals should provide post-exposure counseling to dispel myths regarding the hepatotoxicity of prophylactic drugs. Finally, to improve compliance with prophylactic treatment the recently announced shorter combination regimen should be investigated for its effectiveness, safety, and compliance in China.^15^

## References

[1] Hao Y Epidemics and control strategy of tuberculosis in China. Chin J Tuberc Pespir Dis. 2004;27:433–4 (in Chinese). 15312551

[2] Ministry of Health of the Peoples Republic of China. Guideline of China Tuberculosis Control Program. Beijing: Peking Union Medical College Publishing House; 2008 (in Chinese).

[3] Chen L, Hu Y Investigation of tuberculosis infection among college freshmen in Wuhan. Chin J Gen Pract. 2005;4:424–5 (in Chinese).

[4] Wang S, Qian W, Zheng J, Liu Z Construction of school without tuberculosis disease. J Chin Antituberculosis Assoc. 2008;30 Suppl:S19–20 (in Chinese).

[5] Mazurek GH, Jereb J, Lobue P, Iademarco MF, Metchock B, Vernon A; Division of Tuberculosis Elimination, National Center for HIV, STD, and TB Prevention, Centers for Disease Control and Prevention (CDC) Guidelines for using the QuantiFERON-TB Gold test for detecting Mycobacterium tuberculosis infection, United States. MMWR Recomm Rep. 2005;54 RR-15:49–55 16357824

[6] Nelson K Tuberculin testing to detect latent tuberculosis in developing countries. Epidemiology. 2007;18:348–9 10.1097/01.ede.0000259985.76928.64 17435443

[7] Wang D. Tuberculosis Control and Prevetion. Beijing: China Union Medical College Press; 2004 (in Chinese).

[8] Gustafson P, Lisse I, Gomes V, Vieira CS, Lienhardt C, Nauclér A Jensen H, Risk factors for positive tuberculin skin test in Guinea-Bissau. Epidemiology. 2007;18:340–7 10.1097/01.ede.0000259987.46912.2b 17435442

[9] Phillips L, Carlile J, Smith D Epidemiology of a tuberculosis outbreak in a rural Missouri high school. Pediatrics. 2004;113:e514–9 10.1542/peds.113.6.e514 15173530

[10] Ministry of Health, Ministry of Education, Peoples Republic of China. Standard of Tuberculosis Prevention and Control in Schools. http://www.moh.gov.cn/publicfiles/business/htmlfiles/mohjbyfkzj/s3589/201008/48439.htm [accessed August 6, 2010] (in Chinese).

[11] Washington State Department of Healthy. Washington State Tuberculosis Services Manual. http//:www.doh.wa.gov/cfh/tb/manual/sections/section11.pdf [accessed July 11, 2011].

[12] American Thoracic Society; CDC; Infectious Diseases Society of America Treatment of tuberculosis. MMWR Recomm Rep. 2003;52 RR-11:1–77 12836625

[13] Bi JR, Su HT, Cheng Y, Jin Y Analysis of 127 patients with hepatic lesion due to antituberculosis drugs. Chinese Journal of Antituberculosis. 2002;24:233 (in Chinese)

[14] Fortunato I, Sant’Anna C Screening and follow-up of children exposed to tuberculosis cases, Luanda, Angola. Int J Tuberc Lung Dis. 2011;15:1359–61 10.5588/ijtld.11.0092 22283895

[15] Sterling TR, Villarino ME, Borisov AS, Shang N, Gordin F, Bliven-Sizemore E, TB Trials Consortium PREVENT TB Study Team. Three months of rifapentine and isoniazid for latent tuberculosis infection. N Engl J Med. 2011;365(23):2155–66 10.1056/NEJMoa1104875 22150035

